# Predicting Intracranial Hypertension in Traumatic Brain Injury Using AI: A Systematic Review of Algorithms and Their Clinical Integration Potential

**DOI:** 10.7759/cureus.83267

**Published:** 2025-04-30

**Authors:** Marwa Mohamed Ahmed Elkhidir Babikir, Fatima Ibrahim Abdalla Ibrahim, Haram Hafiz Osman Elhassan, Fatin A Elhaj, Sara Omer Hamid Zain Elabdin, Shahd Abdullahi Sidahmed Mohammed, Manar Eltayeb Mohamed Osman, Dalia Ahmed

**Affiliations:** 1 Internal Medicine, Najran Armed Forces Hospital, Ministry of Defense Health Services, Najran, SAU; 2 Internal Medicine, Dr. Erfan and Bagedo General Hospital, Jeddah, SAU; 3 College of Arts, Sciences, and Information Technology, University of Khorfakkan, Sharjah, ARE; 4 Geriatric Medicine, Stepping Hill Hospital, Stockport NHS Foundation Trust, Stockport, GBR; 5 Internal Medicine, Sheikh Jaber Al-Ahmad Al-Sabah Hospital, Kuwait, KWT; 6 Internal Medicine, SEHA Clinic, Al-Ain, ARE; 7 Care of the Elderly, Barking, Havering and Redbridge University Hospitals NHS Trust, London, GBR

**Keywords:** artificial intelligence, clinical integration, intracranial hypertension, predictive modeling, traumatic brain injury

## Abstract

Intracranial hypertension (ICH) is a critical complication of traumatic brain injury (TBI), associated with poor outcomes. AI shows promise for early ICH prediction, but its clinical integration remains uncertain. This systematic review evaluates the performance, clinical applicability, and limitations of AI models for ICH prediction in TBI. We searched PubMed, Embase, IEEE Xplore, and Scopus, identifying 250 records. After removing duplicates and screening titles and abstracts, 37 full-text articles were assessed, with 9 studies meeting the inclusion criteria. Risk of bias was evaluated using PROBAST, and data on algorithms, performance metrics, and clinical integration were extracted. The included studies demonstrated strong predictive performance, with ensemble models achieving the highest accuracy. However, reliance on invasive monitoring, small sample sizes, and retrospective designs limited generalizability. Only one non-AI study reported clinical integration, highlighting a translational gap. While AI models show potential for ICH prediction, methodological heterogeneity and the lack of prospective validation hinder clinical adoption. Future research should prioritize standardized outcomes, model explainability, and real-world testing to bridge this gap.

## Introduction and background

Traumatic brain injury (TBI) remains a leading cause of morbidity and mortality worldwide, with an estimated 69 million individuals sustaining a TBI annually, contributing significantly to long-term disability and socioeconomic burden [1]. A critical determinant of poor outcomes in TBI is the development of intracranial hypertension (ICH), defined as a sustained elevation in intracranial pressure (ICP) above 20-22 mmHg [2]. ICH exacerbates secondary brain injury by impairing cerebral perfusion, triggering ischemia, and precipitating herniation syndromes [3]. Timely detection and intervention are pivotal to mitigating these cascading insults; however, while conventional methods for monitoring ICP, such as invasive parenchymal or intraventricular catheters, remain the gold standard, they are resource-intensive, carry inherent risks (e.g., infection, hemorrhage), and are not universally accessible, particularly in low-resource settings. Although several validated non-invasive techniques have emerged, their clinical adoption remains variable due to limitations in accuracy, availability, or required expertise [2]. These limitations underscore the urgent need for innovative, non-invasive, and scalable strategies to predict ICH risk and guide precision therapies [4].

In recent years, AI has emerged as a transformative tool in critical care, leveraging multimodal data, including vital signs, neuroimaging, electrophysiological signals, and laboratory parameters, to identify complex patterns predictive of clinical deterioration [5]. Machine learning (ML) and deep learning (DL) algorithms, in particular, offer unparalleled potential to synthesize high-dimensional, time-series data from TBI patients into actionable insights [6]. Early studies suggest that AI models can forecast ICH onset hours before invasive thresholds are breached, enabling preemptive clinical interventions [6]. However, the field remains nascent, with heterogeneous methodologies, variable validation frameworks, and unresolved challenges in translating algorithmic performance into real-world clinical workflows.

This systematic review seeks to synthesize and critically evaluate the current state of AI-driven approaches for predicting ICH in TBI populations. We examine the technical architectures of proposed algorithms, their performance metrics, and the clinical relevance of input features. Furthermore, we assess the translational potential of these models by addressing barriers to clinical integration, including dataset biases, model interpretability, regulatory hurdles, and ethical considerations. By contextualizing algorithmic innovation within the practical demands of neurocritical care, this review aims to bridge the gap between computational research and bedside application, ultimately informing future directions for personalized, AI-enhanced management of TBI.

## Review

Methodology

Study Design

This systematic review was conducted in accordance with the Preferred Reporting Items for Systematic Reviews and Meta-Analyses (PRISMA) guidelines to ensure methodological rigor, transparency, and reproducibility [7].

Search Strategy and Study Selection

A comprehensive literature search was performed across four electronic databases, PubMed, Embase, IEEE Xplore, and Scopus, to identify studies published in English that evaluated AI or ML models for predicting ICH in TBI populations. No publication date restrictions were applied to ensure inclusivity of all relevant studies, given the rapidly evolving nature of AI in healthcare. The search strategy combined Medical Subject Headings (MeSH) terms and keywords related to TBI (“traumatic brain injury,” “head injury”), intracranial hypertension (“intracranial pressure,” “ICP elevation”), and AI/ML techniques (“machine learning,” “deep learning,” “predictive analytics”). Grey literature, including conference proceedings and preprint servers (e.g., arXiv, bioRxiv), was also screened to capture emerging research. The detailed search strategy for each database is listed in Appendix 1.

Initial search results were imported into a reference management software, and duplicates were removed. Two independent reviewers (MHOE & FAE) from the list of authors screened titles and abstracts against predefined inclusion criteria: studies proposing or validating AI/ML models for ICH prediction in TBI, irrespective of study design (observational, retrospective, or prospective). Case reports, editorials, and non-English studies were excluded. Full-text articles of eligible studies were then assessed for final inclusion. Discrepancies between reviewers were resolved through consensus or consultation with a third reviewer (SOHE), who served as a tiebreaker.

Data Extraction

A standardized data extraction template was developed and piloted to ensure consistency across studies. Two reviewers independently extracted the following data from each included study: author(s) and publication year; country and clinical setting; study design (e.g., retrospective cohort, prospective validation); population characteristics (including sample size and TBI severity stratification); AI/ML algorithm type (e.g., logistic regression, support vector machines, neural networks); data input types (e.g., vital signs, imaging data, multimodal biosignals); outcome measures (e.g., ICP thresholds, time-to-event prediction); validation method (e.g., hold-out validation, cross-validation); performance metrics (e.g., sensitivity, specificity, Area under the Receiver Operating Characteristic Curve (AUC-ROC)); reported barriers or facilitators to clinical integration; and key findings. Missing data were requested from corresponding authors via email.

Risk of Bias Assessment

The risk of bias and methodological quality of included studies were evaluated using the Prediction model Risk Of Bias ASsessment Tool (PROBAST) [8]. This tool assesses bias across four domains: (1) participants (selection criteria and representativeness); (2) predictors (definition and measurement); (3) outcome (relevance and ascertainment); and (4) analysis (statistical methods, handling of missing data, and overfitting). Each domain was rated as having “high,” “low,” or “unclear” risk of bias. Two reviewers independently applied PROBAST, with disagreements resolved through discussion. Studies were not excluded based on bias assessment but were critically appraised to contextualize the validity of their conclusions.

Data Synthesis and Analysis

Due to significant heterogeneity in study designs, AI methodologies, outcome definitions, and performance metrics, a meta-analysis was deemed inappropriate. Instead, a narrative synthesis was conducted to categorize findings thematically. Studies were grouped by algorithm type (e.g., traditional ML vs. DL), data inputs (e.g., unimodal vs. multimodal), and clinical applicability (e.g., real-time prediction vs. retrospective risk stratification). Performance metrics were tabulated and compared descriptively, with emphasis on sensitivity (to prioritize early ICH detection) and AUC-ROC (to evaluate overall discriminative ability). Trends in model validation practices, such as the use of external datasets or temporal validation, were analyzed to assess generalizability.

Qualitative Analysis of Clinical Integration

To evaluate the translational potential of AI models, extracted data on clinical integration were analyzed through a qualitative lens. Factors such as feasibility of data acquisition, interoperability with existing hospital systems, clinician interpretability, and ethical considerations were synthesized. Barriers reported across studies, such as small sample sizes or lack of prospective validation, were mapped to stages of the AI development pipeline to propose targeted solutions for future research.

Ethical Considerations and Reporting

This review adhered to ethical standards by ensuring transparency in reporting conflicts of interest and avoiding misrepresentation of study findings. All included studies were cited appropriately, and efforts were made to highlight limitations and biases in the evidence base to prevent overinterpretation of results.

Results

Studies Selection Process

**Figure 1:**
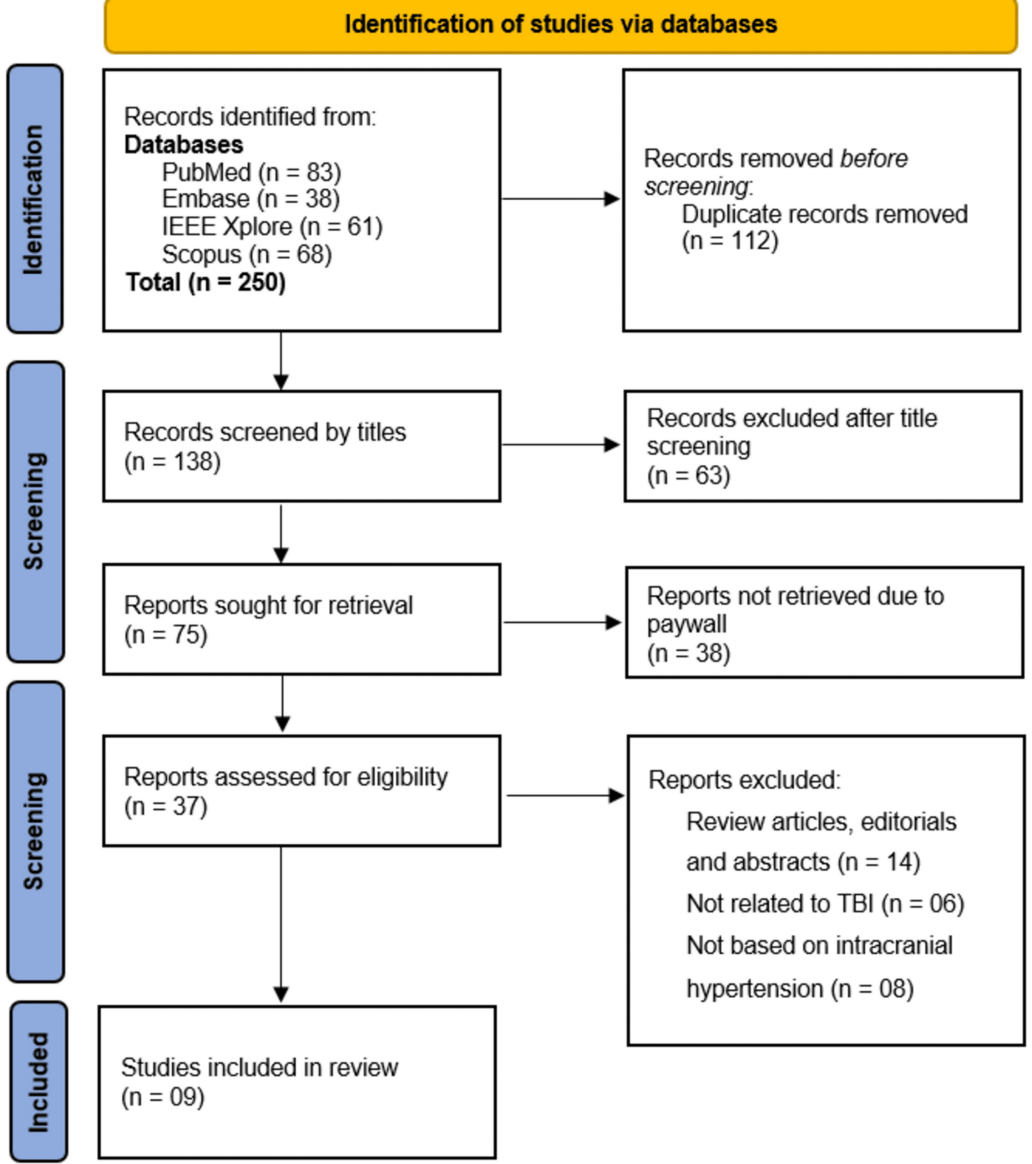
Flowchart of literature identification, screening, eligibility assessment, and inclusion, following PRISMA guidelines. PRISMA: Preferred Reporting Items for Systematic Reviews and Meta-Analyses.

The initial database search identified 250 records from PubMed (n = 83), Embase (n = 38), IEEE Xplore (n = 61), and Scopus (n = 68). After removing 138 duplicates, 112 records were screened by title, excluding 63 irrelevant studies. Of the remaining 75 reports sought for retrieval, 38 were inaccessible due to paywalls. The 37 full-text articles assessed for eligibility excluded 14 review articles/editorials, 6 unrelated to TBI, and 8 not focused on ICH, yielding 9 studies for final inclusion (Figure 1).

Study Characteristics

This systematic review included nine studies [9-17], reflecting a temporal evolution in methodologies and technological advancements in AI-driven ICH prediction. The studies were conducted across diverse clinical settings, including multicenter collaborations in Europe, Level I trauma centers in the United States, and ICUs in Sweden and Singapore. Sample sizes varied widely, ranging from 29 patients [11] to 817 patients [13], with most studies focusing on adults with severe TBI (Glasgow Coma Scale (GCS) < 8). Study designs were predominantly retrospective (n = 6), with three prospective or external validation studies [9, 12]. Two studies included pediatric populations [12], while the rest focused exclusively on adults. Data inputs spanned invasive ICP monitoring, mean arterial pressure (MAP), brain tissue oxygen (PbtO₂), and derived parameters such as pressure reactivity index (PRx). Only one study [14] implemented a non-AI clinical intervention, highlighting the predominance of proof-of-concept AI models in this field (Table 1).

**Table 1:** Characteristics and key findings of included studies. AI: Artificial Intelligence; CRASH: Corticosteroid Randomisation After Significant Head Injury; GCS: Glasgow Coma Scale; GOS: Glasgow Outcome Scale; ICP: Intracranial Pressure; ICU: Intensive Care Unit; ICH: Intracranial Hypertension; IMPACT: International Mission for Prognosis And Clinical Trials in Traumatic Brain Injury; MAP: Mean Arterial Pressure; pbtO₂: Partial Brain Tissue Oxygen Tension; PRx: Pressure Reactivity Index; PROBAST: Prediction model Risk Of Bias ASsessment Tool; TBI: Traumatic Brain Injury; USA: United States of America; UK: United Kingdom.

Author (Year)	Country / Setting	Study Design	Population / Sample Size	AI Algorithm Used	Data Input Type	Outcome Measured	Validation Method	Performance Metrics	Clinical Integration	Key Findings
Carra et al., [9] (2023)	Multi-center; External validation on CENTER-TBI dataset (Europe)	Model development and external validation	290 adult patients (development), 264 patients (validation - CENTER-TBI dataset)	Machine Learning	Minute-by-minute ICP and mean arterial pressure (MAP) signals	Prediction of harmful ICP dose episodes (30 min ahead)	External validation using CENTER-TBI dataset	AUC: 0.94, Accuracy: 0.89, Precision: 0.87, Sensitivity: 0.78, Specificity: 0.94, Calibration-in-the-large: 0.03, Calibration slope: 0.93	Not yet implemented; interventional study needed	ML model accurately predicted harmful ICP doses 30 minutes in advance; potential for proactive TBI management and outcome improvement if integrated.
Petrov et al., [10] (2023)	Level I Trauma Center, United States	Retrospective study	36 patients with severe TBI (GCS < 8)	Random Forest, Light Gradient Boosting, Extreme Gradient Boosting	Continuous ICP time-series data	ICP crisis (ICP > 22 mmHg for ≥75% of a 5-minute interval)	70/30 training/testing split (30 patients), final validation on 5 patients	AUC: 0.86–0.88, Accuracy: 0.82–0.88, Precision: 0.76, Recall: 0.46, F1 Score: 0.57	Potential for integration into clinical workflows	Model predicted ICP crises 10–20 min in advance with strong accuracy; can aid in early interventions and reduce secondary brain injury in severe TBI patients.
Wijayatunga et al., [11] (2022)	ICU setting, Sweden	Model development / validation	29 patients with severe TBI	Probabilistic Markov model	ICP time series (past hour)	Prediction of ICP ≥ 20 mmHg	80/20 random split & leave-one-out cross-validation	Specificity: 0.90–0.95; Sensitivity: 0.73–0.89	Not yet integrated; planned for future evaluation	The model accurately predicted elevated ICP levels. Enhancement method improved results. Model is expandable with other data types. Suitable for future clinical use.
Güiza et al., [12] (2017)	Multicenter: Italy (San Gerardo Hospital), Belgium (Leuven & Antwerp University Hospitals), Germany (Tübingen), UK (Glasgow, Edinburgh, Newcastle)	Prognostic modeling; non-interventional, observational, retrospective study	Adults: n=121; Pediatrics: n=79	Not specified	Intracranial pressure (ICP) and mean arterial pressure (MAP)	Early detection of increased ICP episodes	External validation on new adult and pediatric cohorts	Discrimination, calibration, overall performance, clinical usefulness	Not yet integrated, but step toward early warning system	Model showed excellent performance in adults and good discrimination in pediatrics; confirms robustness for predicting increased ICP 30 min in advance using only 2 input signals.
Myers et al., [13] (2016)	USA / Ben Taub Hospital, Houston, TX	Retrospective study	817 patients with severe TBI	Multivariate classification	Physiologic signals, ICP, and time since last crisis	Prediction of ICP ≥ 20 mmHg (≥15 min) and PbtO₂ < 10 mmHg (≥10 min)	Not specified	AUC = 0.86 for ICP prediction; AUC = 0.91 for PbtO₂ prediction	Not integrated yet (research phase)	Algorithms predict ICP and PbtO₂ crises up to 30 minutes in advance with high accuracy using limited physiologic inputs (ICP and time since last crisis).
Beckers et al., [14] (2014)	UK / Queen’s Hospital, Romford	Audit (before-after study)	240 prescriptions (129 pre, 111 post)	None	Prescription charts	Prescribing accuracy and error reduction	Repeat audit post-intervention	70% improvement in safe prescribing; 94% met best practice post	Introduced standardized prescription stickers and education	Standardized tools and education improved safe prescribing from 24% to 94% adherence; cost-effective (£20 for 6,200 stickers); reduced undocumented infusions.
Güiza et al., [15] (2013)	22 neuro-ICUs in 11 European countries	Prognostic modeling; non-interventional, retrospective observational study	264 traumatic brain injury patients	Multivariate logistic regression and Gaussian processes	Time-series summary statistics of minute-by-minute MAP and ICP; static admission data (CRASH-basic and IMPACT-core predictors)	1. Prediction of increased ICP episodes (30 min ahead); 2. Prediction of poor neurologic outcome at 6 months (GOS 1–2 and 1–3)	External validation using a separate dataset	ICP prediction AUC = 0.87; GOS 1–2 prediction AUC improved from 0.72 to 0.90; GOS 1–3 prediction improved from 0.68 to 0.87	Not explicitly integrated in clinical practice (research phase)	Dynamic monitoring data significantly improves prediction of both acute secondary injury (ICP) and long-term neurologic outcomes in TBI patients. ICP episodes predicted 30 mins in advance with good calibration.
Feng et al., [16] (2012)	Singapore	Retrospective observational study	82 TBI patients monitored (2002–2007)	Temporal vs. non-temporal forecasting models	Time-series data of ICP, MAP, PbtO₂, and calculated PRx	Forecasting trends in ICP and related parameters	Paired t-test (statistical comparison)	20% average performance gain (temporal vs. non-temporal), p < 0.0001	Not reported	Temporal models outperformed non-temporal models in trend forecasting; results statistically significant with ~20% performance gain.
Klauber et al., [17] (1984)	United States	Retrospective cohort	Severely head-injured patients	Logistic Regression	Clinical variables during first 24 hours	Delayed intracranial hypertension (ICP > 30 mmHg)	Separate cross-validation group	Error rate: 24% (initial), 20% (validation); Sensitivity: 86–89%	Not explicitly stated	Peak ICP during first 24 hrs was the strongest predictor; model used additional predictors like hypotension and abnormal ventricles.

Algorithmic Approaches and Predictive Performance

ML algorithms demonstrated strong discriminatory performance in predicting ICH, with AUC-ROC values ranging from 0.72 to 0.94. Ensemble methods, such as Random Forest (RF), Light Gradient Boosting Machine (LGBM), and Extreme Gradient Boosting (XGBoost), achieved the highest performance metrics. For example, Petrov et al. [10] reported AUCs ranging from 0.86 to 0.88 (95% CI: 0.82-0.91) using ensemble models to predict ICP crises 10-20 minutes in advance. Similarly, Carra et al. [9] achieved an AUC of 0.94 with external validation on a multicenter dataset, demonstrating generalizability across heterogeneous populations. However, sensitivity varied significantly across studies (0.46-0.89), with lower recall in models prioritizing specificity (e.g., 0.46 recall in Petrov et al. [10]). Probabilistic approaches, such as the Markov model by Wijayatunga et al. [11], balanced specificity (0.90-0.95) and sensitivity (0.73-0.89) but were limited by small sample sizes.

Simpler models, including logistic regression [15, 17] and Gaussian processes [12], showed moderate performance (AUC 0.72-0.87) but offered advantages in interpretability and clinical feasibility. Temporal forecasting models [16] outperformed non-temporal approaches by 20% in trend prediction, emphasizing the value of time-series data. Notably, models predicting secondary outcomes, such as 6-month neurologic prognosis [15], achieved AUC improvements from 0.68 to 0.90 when incorporating dynamic ICU data.

Clinical Integration and Validation Frameworks

Despite strong predictive performance, none of the AI models have been prospectively integrated into clinical workflows. Validation methods were heterogeneous: four studies employed external validation [9, 12-14], while others used hold-out splits [10] or cross-validation [11]. Models relying on high-frequency, multimodal data (e.g., continuous ICP waveforms) faced practical barriers, such as interoperability with existing hospital systems and the need for invasive monitoring. By contrast, simpler models using static admission data [17] or limited physiologic inputs (ICP + MAP) demonstrated greater feasibility but sacrificed predictive granularity.

Risk of Bias Assessment Results

The risk of bias assessment using the PROBAST tool revealed significant variability across the included studies. Two studies [9, 15] demonstrated low overall risk of bias due to robust participant selection, well-defined predictors and outcomes, and appropriate analytical methods, including external validation and calibration. However, five studies [10, 11, 13, 14, 17] were rated as having high overall risk, primarily due to selection bias (e.g., single-center cohorts, small sample sizes), inadequate handling of missing data, or overfitting in model development. Studies such as Güiza et al. [12] and Feng et al. [16] were deemed to have unclear overall risk, owing to insufficient details on predictor measurement or participant recruitment. Common methodological limitations included retrospective designs prone to confounding, lack of clarity in outcome ascertainment, and insufficient external validation, particularly in older studies [17]. These findings underscore the need for improved transparency in reporting and adherence to rigorous validation frameworks to enhance the reliability of AI models in predicting ICH (Table 2).

**Table 2:** Risk of bias assessment using PROBAST tool. PROBAST: Prediction model Risk Of Bias ASsessment Tool.

Study (Year)	Participants	Predictors	Outcome	Analysis	Overall Risk
Carra et al. [9] (2023)	Low	Low	Low	Low	Low
Petrov et al. [10] (2023)	High	Low	High	High	High
Wijayatunga et al. [11] (2022)	High	Low	Unclear	Unclear	High
Güiza et al. [12] (2017)	Low	Unclear	Low	Low	Unclear
Myers et al. [13] (2016)	Low	Low	High	High	High
Beckers et al. [14] (2014)	Unclear	High	Low	High	High
Güiza et al. [15] (2013)	Low	Low	Low	Low	Low
Feng et al. [16] (2012)	Unclear	Low	Low	Low	Unclear
Klauber et al. [17] (1984)	High	Low	Unclear	Unclear	High

Comparative Analysis of AI Models for ICH Prediction in TBI

Table 3 highlights essential features such as the type of algorithm used, prediction horizon, data input complexity, readiness for clinical integration, and key advantages and limitations. By synthesizing these elements, this comparative analysis aids in recognizing common themes, identifying gaps in translational progress, and outlining directions for future research in AI applications for ICH prediction in TBI.

**Table 3:** Comparative summary of AI models predicting intracranial hypertension in TBI. AI: Artificial Intelligence; RF: Random Forest; LGBM: Light Gradient Boosting Machine; XGBoost: Extreme Gradient Boosting; ML: Machine Learning; ICP: Intracranial Pressure; MAP: Mean Arterial Pressure; PbtO₂: Partial Brain Tissue Oxygen Tension; PRx: Pressure Reactivity Index.

Study (Author, Year)	AI Approach	Prediction Horizon	Input Complexity	Clinical Readiness	Notable Strengths	Key Limitations
Carra et al., [9] (2023)	Machine Learning	30 min ahead	Moderate (ICP + MAP)	Not integrated	High accuracy (AUC 0.94); external validation	Needs prospective validation
Petrov et al., [10] (2023)	Ensemble ML (RF, LGBM, XGBoost)	10-20 min ahead	High (continuous ICP)	Potential for integration	Strong accuracy in crisis prediction	Small external validation sample
Wijayatunga et al., [11] (2022)	Probabilistic Markov model	Short-term prediction	Moderate (ICP time series)	Not integrated	Expandable model; strong specificity	Small sample size
Güiza et al., [12] (2017)	Not specified (ML framework)	30 min ahead	Low (ICP + MAP)	Not integrated	Pediatric + adult validation; simple inputs	Algorithm transparency not described
Myers et al., [13] (2016)	Multivariate classification	30 min ahead	Moderate (physiologic + ICP)	Research phase	Simultaneous ICP and PbtO₂ prediction	Clinical pathway not defined
Beckers et al., [14] (2014)	None (audit-based study)	Not predictive (intervention impact)	Low (prescription data)	Integrated (audit context)	Improved safe prescribing through standardization	Not an AI-based ICP prediction study
Güiza et al., [15] (2013)	Logistic regression + Gaussian processes	30 min ahead + 6-month outcomes	High (dynamic + static data)	Not integrated	Acute + long-term outcome prediction using ICU data	Complex model; requires high data granularity
Feng et al., [16] (2012)	Temporal forecasting ML	Trend-based (not fixed)	High (ICP, MAP, PRx)	Not reported	Significant improvement over non-temporal models	No clear clinical link
Klauber et al., [17] (1984)	Logistic regression	Delayed ICP episodes	Low (clinical data, early phase)	Not stated	Historical value; simple predictors	Outdated model; lacks validation with current data

Most models demonstrate strong predictive performance, particularly those utilizing ensemble learning or probabilistic modeling. However, many rely on complex, high-frequency physiological inputs that may hinder seamless clinical adoption. While studies like Carra et al. [9] and Petrov et al. [10] achieved high AUCs and sensitivity, their lack of real-time deployment or prospective evaluation limits immediate impact. Simpler models such as those by Güiza et al. [12] and Klauber et al. [17] underscore the trade-off between data simplicity and predictive power. Notably, only Beckers et al. [14] involved clinical integration, although not AI-based, highlighting a broader implementation gap across AI-driven studies. Overall, this comparative analysis emphasizes the need for more robust validation, user-friendly models, and interdisciplinary collaborations to transition from proof-of-concept to bedside impact.

Discussion

The findings of this systematic review underscore AI’s potential to enhance early detection of ICH, a critical determinant of secondary brain injury and poor outcomes in TBI. However, the review also highlights persistent barriers to clinical translation, including methodological heterogeneity, biases in study design, and unresolved practical challenges in deploying AI tools at the bedside. The studies included in this review demonstrate that ML models, particularly ensemble methods (e.g., Random Forest, Gradient Boosting) and probabilistic frameworks, achieve strong discriminative performance in predicting ICH episodes. AUC-ROC values ranged from 0.72 to 0.94, with ensemble models such as those by Carra et al. [9] and Petrov et al. [10] achieving the highest scores (AUC 0.86-0.94). These results align with advancements in other critical care domains, such as sepsis and cardiac arrest prediction, where ensemble learning and temporal modeling have become cornerstones of predictive analytics. For instance, the integration of multiple algorithms in ensemble approaches mitigates overfitting and enhances robustness against noisy physiological data, a feature particularly relevant in TBI care, where signal artifacts from invasive monitors are common [18].

However, the clinical relevance of these models is tempered by trade-offs between accuracy and feasibility. Models relying on high-frequency, multimodal inputs, such as continuous ICP waveforms, MAP, and pressure reactivity index (PRx), achieved superior predictive granularity [13, 16]. Yet their dependence on invasive monitoring and computational infrastructure limits scalability, particularly in resource-limited settings where invasive ICP sensors are unavailable. By contrast, simpler models using static variables or minimal physiological inputs (e.g., ICP + MAP) prioritized interpretability and ease of implementation but exhibited lower AUCs (0.72-0.87). For example, Klauber et al. [17] demonstrated that logistic regression using first-day clinical variables (e.g., peak ICP, hypotension) could predict delayed ICH with 86-89% sensitivity, albeit in an outdated cohort. This dichotomy underscores a fundamental challenge in medical AI: balancing precision with practicality.

The superior performance of temporal models further emphasizes the importance of time-series data in ICH prediction. Feng et al. [16] reported a 20% improvement in trend forecasting when incorporating temporal dynamics, a finding consistent with studies in hemodynamic instability prediction. Time-aware architectures, such as recurrent neural networks (RNNs) or long short-term memory (LSTM) networks, could further enhance predictive horizons by capturing latent patterns in physiological deterioration [19]. However, the absence of such advanced architectures in the reviewed studies suggests a lag in adopting cutting-edge AI techniques in TBI research.

Despite high AUCs and sensitivity metrics, none of the studies progressed beyond retrospective validation or small-scale prototyping. This stands in stark contrast to non-AI interventions, such as the audit-based protocol by Beckers et al. [14], which achieved 94% adherence to safe prescribing practices through workflow standardization. The disparity highlights a systemic issue in medical AI development: while models excel in controlled environments, their translation to real-world settings is hampered by interoperability challenges, clinician skepticism, and insufficient usability testing.

Models requiring continuous, high-resolution data inputs [10] assume universal access to invasive monitors and interoperable electronic health record (EHR) systems, a presumption at odds with global healthcare realities. In low-resource ICUs, where invasive ICP monitoring is rare, such models are clinically irrelevant. Even in well-resourced settings, interoperability barriers persist. For instance, integrating AI alerts into existing EHR platforms requires seamless data pipelines, middleware solutions, and compliance with regulatory standards [20], challenges seldom addressed in the reviewed studies. The lack of cost-effectiveness analyses further obscures the feasibility of scaling these tools.

The "black-box" nature of complex AI models erodes clinician trust, a barrier exacerbated by the absence of explainability frameworks in most studies. While ensemble models like XGBoost offer intrinsic feature importance metrics, few studies [11] incorporated post-hoc interpretability tools such as SHapley Additive exPlanations (SHAP) or Local Interpretable Model-agnostic Explanations (LIME). Clinicians require transparent rationale for AI-driven alerts to justify high-stakes interventions, such as escalating sedation or performing decompressive craniectomy. Without explainability, even high-performing models risk being dismissed as impractical.

The reviewed studies largely overlooked ethical challenges, including algorithmic bias and equity in access. For example, models trained on homogeneous cohorts (e.g., single-center studies in high-income countries) may underperform in underrepresented populations, such as pediatric TBI patients or those with comorbid conditions. Güiza et al. [12] made strides in external validation across adult and pediatric cohorts, but such efforts remain exceptions. Additionally, the reliance on invasive monitoring perpetuates a "digital divide," excluding patients in settings where ICP sensors are unavailable. These gaps mirror broader critiques of AI in global health, where technological advancements often exacerbate inequities.

Only two studies [9, 15] reported calibration metrics, a critical oversight, given that poorly calibrated models, even with high AUCs, may yield unreliable probability estimates. For example, a model predicting a 90% risk of ICH with poor calibration could systematically over- or underestimate true probabilities, leading to inappropriate clinical actions. Overfitting further compounds this issue, particularly in studies using high-dimensional data without regularization [11]. Cross-validation, while employed in some studies, is insufficient to address overfitting in small datasets.

The lack of standardized outcome definitions (e.g., varying ICP thresholds, crisis durations) complicates cross-study comparisons. While most studies defined ICH as ICP > 20-22 mmHg, crisis durations ranged from 5-minute intervals [10] to 30-minute windows [9]. Such variability undermines efforts to benchmark performance or synthesize evidence. Additionally, few studies evaluated downstream clinical outcomes (e.g., mortality, functional status), focusing instead on surrogate endpoints like ICP thresholds. This misalignment with patient-centered outcomes limits the perceived value of AI tools among clinicians.

Future directions

Future studies must prioritize prospective validation in diverse, multicenter cohorts. The success of Carra et al. [9] in externally validating their model using the CENTER-TBI dataset exemplifies this approach. Real-world testing should also assess integration into clinical workflows, measuring outcomes such as alert fatigue, clinician compliance, and time-to-intervention. Standardizing outcome definitions (e.g., adopting the Seattle International Severe Traumatic Brain Injury Consensus Guidelines) and performance metrics (e.g., mandating calibration plots) would enhance comparability. Collaborative frameworks, such as the TBI Endpoints Development Initiative, could facilitate data pooling and improve model generalizability.

Incorporating explainability tools and involving clinicians in model design are imperative. Hybrid models that combine ML with rule-based logic (e.g., “if ICP > 22 mmHg for 5 minutes, then alert”) may enhance trust while preserving accuracy. User-centered design studies, though absent in this review, are critical to refining AI interfaces for busy ICU environments. Efforts to develop non-invasive predictors (e.g., transcranial Doppler, pupillometry) must be accelerated to democratize access. Partnerships with low-resource settings can ensure models are trained on diverse populations, addressing algorithmic bias and improving global relevance.

Limitations

This review has several limitations. First, heterogeneity in study designs, outcome definitions, performance metrics, and statistical approaches used to evaluate prognostic performance, such as differing applications of ROC curves and AI metrics across studies, may introduce bias and complicate comparative interpretation. Second, the exclusion of non-English studies and grey literature (beyond preprints) may have omitted innovative approaches. Third, the focus on peer-reviewed articles likely overlooks ongoing industry-led projects or clinical trials. Finally, the lack of cost-effectiveness data in the included studies restricted analysis of economic feasibility, a critical factor for healthcare administrators.

## Conclusions

AI holds immense potential to revolutionize ICH prediction in TBI, offering a lifeline for mitigating secondary brain injury. However, the field remains in its infancy, constrained by methodological inconsistencies, biases, and a glaring implementation gap. Bridging this gap demands a paradigm shift, from isolated model development to interdisciplinary collaboration, rigorous prospective validation, and an unwavering focus on equity. By addressing these challenges, researchers can transform AI from a promising tool into a cornerstone of precision neurocritical care, ensuring that advancements benefit all patients, regardless of geography or resource constraints. The journey from proof of concept to bedside impact is arduous, but the stakes, for patients, clinicians, and healthcare systems, could not be higher.

## Appendices

Appendix 1 

**Table 4:** Detailed search strategy for each database used in this study.

Sr. No	Database	Search String
1	PubMed	("Intracranial Hypertension"[Mesh] OR "intracranial pressure" OR "intracranial hypertension" OR "raised intracranial pressure") AND ("Brain Injuries, Traumatic"[Mesh] OR "traumatic brain injury" OR "TBI") AND ("Artificial Intelligence"[Mesh] OR "Machine Learning"[Mesh] OR "Deep Learning" OR "neural network*" OR "AI" OR "algorithm*" OR "predictive model*" OR "computer-aided" OR "automated detection") AND ("Prediction" OR "Prognosis" OR "diagnosis" OR "monitoring" OR "clinical decision support")
2	Embase	('intracranial hypertension'/exp OR 'intracranial pressure' OR 'raised intracranial pressure') AND ('traumatic brain injury'/exp OR 'brain injury, traumatic' OR 'TBI') AND ('artificial intelligence'/exp OR 'machine learning'/exp OR 'deep learning'/exp OR 'neural network'/exp OR 'AI' OR 'algorithm' OR 'predictive model' OR 'automated detection') AND ('prediction' OR 'prognosis' OR 'monitoring' OR 'diagnosis' OR 'clinical decision support')
3	IEEE Xplore	(("intracranial pressure" OR "intracranial hypertension" OR "raised ICP") AND ("traumatic brain injury" OR "TBI") AND ("artificial intelligence" OR "machine learning" OR "deep learning" OR "neural network" OR "algorithm" OR "predictive model" OR "AI") AND ("prediction" OR "monitoring" OR "prognosis" OR "diagnosis" OR "clinical decision support"))
4	Scopus	(TITLE-ABS-KEY("intracranial hypertension" OR "intracranial pressure" OR "raised intracranial pressure")) AND (TITLE-ABS-KEY("traumatic brain injury" OR "TBI")) AND (TITLE-ABS-KEY("artificial intelligence" OR "machine learning" OR "deep learning" OR "neural network*" OR "AI" OR "algorithm*" OR "predictive model*" OR "automated detection")) AND (TITLE-ABS-KEY("prediction" OR "prognosis" OR "monitoring" OR "diagnosis" OR "clinical decision support"))
